# Supplementary material for: Most Common Long COVID Physical Symptoms in Working Age Adults Who Experienced Mild COVID-19 Infection: A Scoping Review
Source: Healthcare (Basel). 2022 Dec 19;10(12):2577. doi: 10.3390/healthcare10122577 (PMC9778298; doi:10.3390/healthcare10122577)
Supplement: Supplementary file 1 [file healthcare-10-02577-s001.zip › healthcare-2033250-supplementary.pdf]

Supplementary Table 1:

All relevant data including the author, year, and country, aims of the study, methods and research design, sample size, and the most common long COVID physical symptoms.

Author, Country, Year	Aim(s) of the study	Study Design	Data Collection protocol/ collection tool	Timeline of Data collection	Sample Size	Most common Symptoms	Days post infection	Average age	% of Women	AverageBMI	Smokers
1.Aly, et al, Egypt 2021	To investigate post-COVID-19 symptoms amongst elderly females and whether they could be a risk factor for developing chronic fatigue syndrome	Retrospective cross-sectional study	Online survey	September till mid-October 2020	115	Fatigue Dyspnoea, Cough, chest pain, myalgia, arthralgia weakness Headache and dizziness	up 120 days	73 mean	100	Not reported (NR)	47.8
2.Augustin, et al, Germany, 2021	To explore the incidence, diagnostic criteria, and management of long-term health consequences at 4 and 7 months after mild courses of COVID-19	Prospective and longitudinal study	Questionnaire	April 2020-January 2021	442	Fatigue, Dyspnoea, anosmia, muscle and joint pain, cough	median 204 days	Median age at the first visit was 43 years (3154)	53.50%	NR	NR
3.Bakilan, et a Turkey, 2021	To evaluate the most frequent admission symptoms and the frequency of musculoskeletal symptoms in post-acute COVID-19 patients; and- to determine the related factors with the post-acute COVID-19 musculoskeletal symptoms.	Cross-sectional study	EHR	December 2020 and May 2021	240	Fatigue Spine pain, Neck pain, Back pain, Low back pain, Spine pain, Muscle pain, Arthralgia joint pain, Numbness, dyspnoea,	NR	47 Median (39-58)	66.60%	27.55	16.60%

Author, Country, Year	Aim(s) of the study	Study Design	Data Collection protocol/ collection tool	Timeline of Data collection	Sample Size	Most common Symptoms	Days post infection	Average age	% of Women	AverageBMI	Smokers
						Cough, chest pain					
4.Barizien,et al, France, 2021	To demonstrate this phenomenon by illustrating Heart rate variability dysregulation reflected through the NOL index showing the sympathetic/parasympathetic balance, in long COVID-19 participants.	Cross-sectional descriptive study	NOL (nociception level) index measurements, PCL-5, NIJMEGEN	December 2020 to January 2021	27	fatigue	45 days	48 median	47.10%	25.2	NR
5.Bastola, et al, Nepal, 2021	d to study persistent symptoms in post-COVID-19 patients attending a follow-up clinic	Descriptive, cross-sectional study	standardized structured questionnaire	1 March 2021 to 14 April 2021	118	Shortness of breath, Fatigue, Chest heaviness, Cough, Chest pain, Anxiety, Palpitations, Headache	28 days	49.7 Mean +/- 15.01	31.40%	NA	NR
6. Bierle, et al, USA, 2021	To develop and implement criteria for description of post COVID syndrome	Prospective study	DELPHI methods by expert panel	November 15, 2019, to May 8 2020	42	pain, fatigue, dyspnoea, and orthostatic intolerance	77 days	46.2 median 21-46	66.60%	NR	NR
7. Bell et al, USA, 2021	To investigate the impacts of the SARS-Cov-2 pandemic among Arizona residents who were non hospitalized	Prospective cohort study	Online survey	30 days from 28 May 2020 to 24 February 2021	208	Fatigue, shortness of-breath, Fatigue, Shortness of breath, Body aches or muscle pains, Headaches,	60 days	mean age of 44 years (range 12–82 years	70%	28.5kg/m2 (7.3)	27%

Author, Country, Year	Aim(s) of the study	Study Design	Data Collection protocol/ collection tool	Timeline of Data collection	Sample Size	Most common Symptoms	Days post infection	Average age	% of Women	Average BMI	Smokers
						Faster than normal heart rate (Tachycardia), Dizziness/light headedness, Chest pain or tightness, brain fog and anxiety					
8. Bliddal et al, Denmark, 2021	To determine the prevalence of and risk factors for acute and persistent symptoms in non-hospitalized patients	Cohort/ case series (no comparison grp)	Questionnaire	June 24th and August 15th 2020	129	Fatigue, Dyspnoea, Cough, chest pain, Headache, myalgia or arthralgia	84 days	47.4 mean SD 15.2	76.4%	26.8	16.7% (ever)
9. Blomberg et al, Norway, 2021	To identify factors and biomarkers associated with long-term complications, 6 months after initial COVID-19 in a prospective cohort of hospitalized and home-isolated patients	Prospective cohort study	Questionnaire/ blood samples	28 February to 4 April 2020	247	Fatigue, Disturbed taste/smell, Concentration problems, Memory problems, Dyspnoea, Headache, Dizziness	60 days/ 180 days	Median 43 (27–55)	53%	Median 24.3 (22.5–26.5)	NR
10. Boesl et al, Germany, 2021	To report on the clinical presentation of the first 100 patients who presented to our PCS Neurology outpatient clinic ≥12 weeks after the	Descriptive study	ESS, BDI, FSS, MoCA	September 2020 and April 2021	100	Fatigue, joint pain, myalgia, headache, vertigo	184.5 days	45.8 mean (20 – 79)	67%	NR	24.2%moderate or sever

Author, Country, Year	Aim(s) of the study	Study Design	Data Collection protocol/ collection tool	Timeline of Data collection	Sample Size	Most common Symptoms	Days post infection	Average age	% of Women	AverageBMI	Smokers
	acute infection with SARS-CoV-2.										
11. Buoite Stella, et al, Italy, 2021	To assess the prevalence of ANS dysfunction, evaluated with the COMPASS-31 questionnaire and an active stand test, in a real-life setting in consecutive patients referred to the post-COVID ambulatory service, and to compare the patients who presented neurological symptoms with those without neurological manifestations	Prospective observational study,	Clinical and instrumental evaluation by a neurologist, COMPASS-31 questionnaire, active stand test	February 15 to May 15 2021	180	Exertional dyspnoea, Brain fog, Joint pain, Muscle pain and weakness, dizziness	Median 59 days (31-175 days)	51 +/- 13	70.6%	NR	NR
12. Danesh, et al, USA, 2021	To describe the sequelae of COVID-19 in patients referred to COVID recovery clinic services. 2. Examine the similarities of COVID-19 sequelae in the context of initial onset and severity of acute illness in patients seeking COVID recovery care	Cross sectional Observational Study	semi structured interview, EHR	November 2020–February 2021	63	Fatigue, dyspnoea, cough, myalgia and arthralgia, headache, unable to work	median 82 day	52	71%	32% comorbidity was obesity	NR
13. Davin-Casalena, et al, France, 2021	To identify the proportion of French GPs with patients with potential long COVID syndrome and the symptoms they reported	cross-sectional	Online questionnaire	October 6 and November 15, 2020, w	651	Respiratory problems, Headache, Chest pains, sleep disorders	60 – 90 days	NR	40.30%	NR	NR
14. Davis, et al, USA, UK, 2021	To better describe the patient experience and recovery process in those with	Prospective observational study	Survey	September 6, 2020, to	3762	fatigue 70.4%, breathing issues 59.5%	180 days	30 – 60 33.7% betwee	78.80%	NR	NR

Author, Country, Year	Aim(s) of the study	Study Design	Data Collection protocol/ collection tool	Timeline of Data collection	Sample Size	Most common Symptoms	Days post infection	Average age	% of Women	AverageBMI	Smokers
	confirmed or suspected COVID-19 illness, with a specific emphasis on the Long COVID			November 25, 2020.				n ages 40-49 biggest group			
15. Dennis, et al, UK, 2021	investigate (1) the prevalence of multiorgan impairment, compared with healthy, age-matched controls; (2) the associations between typical COVID-19 symptoms and multiorgan impairment; and (3) the associations between hospitalisation, severity of symptoms and multiorgan impairment	Prospective observational cohort study	Questionnaires, Dyspnoea-12,EQ-5D-5L,MRI	1 April 2020 and 14 September 2020,	163	Fatigue, Shortness of breath, muscle ache, Headache, Joint pain, Chest pain, cough, Fever, sore throat	median of 141 days after initial symptoms (range between 110 - 162 days)	43 +/- 10.9	72.4%	25.3% (22.7-27.7)	30%
16. Estiri, et al, USA, 2021	To identify accurate identification of Post-acute Covid sequaele phenotypes in age/ gender groups at two windows of time	Retrospective Cohort Study	EHR and clinical expertise,	March 2020 and June 2021	96025	Fatigue, insomnia, dysgeusia, shortness of breath, chronic fatigue syndrome, and non-specific chest pain	90 – 180 days/ 180 – 270 days	44	71%	median BMI was 25.7	3%
17. Gaber,et al, UK, 2021	To investigate of long-term impact of COVID-19 in Health caseworkers	Prospective Observational Study	Survey monkey questionnaire	July 1st - August 1st	61	fatigue, SOB, sleep disturbances, mood disturbances	90 – 120 days	NR	45%	NR	NR

Author, Country, Year	Aim(s) of the study	Study Design	Data Collection protocol/ collection tool	Timeline of Data collection	Sample Size	Most common Symptoms	Days post infection	Average age	% of Women	AverageBMI	Smokers
18. Ganesh, et al, USA, 2021	To add insight into our understanding of certain post-acute conditions and clinical findings.	Retrospective cross-sectional Study	EMR, PROMIS questionnaire	March 1st , 2020, and September 12th , 2020.	817	Fatigue 16.2%, pain 17.8%, physical function 10.6%, social roles 43.2%, sleep 9.9% disturbance of function	68.4 days	44 mean	61.10%	NR	NR
19. Gonzalez-Andrade, F. Et al, Ecuador, 2021	To identify and compare post-COVID symptoms between 3 groups of outpatients who had experienced mild, moderate, and severe infection and look at risk factors	Retrospective epidemiological + Cross sectional (Symptoms past 7 days)	demographic data, CFQ-11, subjective perception scale	January and April 2021	566	Fatigue, dyspnoea, cough, sputum, chest tightness, chest pain, Palpitations, head ache, sleep disorders, dizziness, Myalgia, Arthralgia, Reduced mobility (inability to move fully or to control movement)	>28 days	39	49.71%	NR	NR
20. Goertz, et al, Netherlands, 2020	To assess whether symptoms recover following the onset of COVID-19	Prospective observational Study	online survey	4–11 June 2020	2113	Fatigue, headache, chest tightness, muscle pain, dizziness, joint paint Dizziness, heart	79 days after	age 43	91%	26 median (23.3-29.4)	NR

Author, Country, Year	Aim(s) of the study	Study Design	Data Collection protocol/ collection tool	Timeline of Data collection	Sample Size	Most common Symptoms	Days post infection	Average age	% of Women	AverageBMI	Smokers
						palpitations, increased resting heart rate					
21. Graham, et al, USA, 2021	To characterize the spectrum of neurologic manifestations in non-hospitalized Covid-19 “long haulers”	Prospective observational	Medical records, PROMIS, NIH Toolbox	February 2020 or later,	100	Fatigue, “brain fog”, headache, numbness/tingling, Myalgia, Dizziness, Pain other than chest, Depression/Anxiety, SOB, Chest pain, Insomnia, variations in HR and BP	mean 30 days	47 median (37-53.5)	70%	25.4	NR
22. Heightman, et al, UK, 2021	To report the baseline characteristics, clinical presentation, management, and outcomes of PACS over a 12-month period	Observational analysis	Post-COVID-19 assessment	April 2020 and April 2021,	566	Breathlessness, fatigue, cough, muscle ache, chest pain, Myalgia, Headache	mean 194 days	44.6 median (35.6 – 52.8)	68.20%	NR	NR
23. Herck, et al, Netherlands, 2021	To assess the severity of fatigue over time in members of online long COVID peer support groups, and to assess whether members of these groups experienced mental fatigue, physical fatigue, or both.	Prospective study	Online survey	June 4 and June 11, 2020 (	239	Fatigue	90 and 180 days	50	82.80%	26 median (23.4 – 30.5)	NR

Author, Country, Year	Aim(s) of the study	Study Design	Data Collection protocol/ collection tool	Timeline of Data collection	Sample Size	Most common Symptoms	Days post infection	Average age	% of Women	AverageBMI	Smokers
24. Holmes, et al, Australia, 2021	To develop the basis of an objective metabolic framework for measuring systemic recovery in COVID-19 patients using a range of metabolic technologies and biomarkers	Prospective cohort study	survey		27	At 3 months: Fatigue, cough, (myalgia) muscle ache, Join pain, chest pain. At 6 months: Fatigue, cough, myalgia, Join pain, chest pain.	90 and 180 days	NR	NR	NR	NR
25. Hossain, et al, Bangladesh, 2021	To identify the prevalence of long COVID symptoms in a large cohort of people living with and affected by long COVID and identify any potential associated risk factors.	Prospective observational study	Survey	June and November 2020	356	Fatigue, cough, myalgia, chest pain	30 – 120 days/ 120 – 217 days	38.7 +/- 11	32%	NR	NR
26. Jacobson et al, USA, 2021	To assess the prevalence of persistent functional impairment after coronavirus disease (COVID-19)	Retrospective study	physical exam, survey of symptoms, WPAI, 6-minute walk test	November to December 2020	49	Fatigue, breathlessness, cough, myalgia, chest pain	90 – 120 days-	41.6 SD =12.5	49%	30 +/-6.1	NR
27. Kamal, et al, Egypt, 2021	To investigate and characterize the manifestations which appear after eradication of the coronavirus infection and its relation to disease severity	Cross sectional observation lstudy	Questionnaire	April and June, 2020	287	fatigue, anxiety, joints pain, continuous headache, chest pain, dementia,	NR	32.3 +/-8.5	64.10%	28.5 +/-5.2	27.2% males were smokers no women

Author, Country, Year	Aim(s) of the study	Study Design	Data Collection protocol/ collection tool	Timeline of Data collection	Sample Size	Most common Symptoms	Days post infection	Average age	% of Women	AverageBMI	Smokers
						depression, and dyspnea					
28. Kashif, et al, Pakistan, 2021	To identify the presence of post-viral symptomatology in patients recovered from mild COVID-19 disease.	Prospective Observational Study	a telephonic follow-up with open-ended questions		242	Fatigue, Myalgia, Sleep disturbances, Chest pain , Cough	90 days	33.65 +/- 11.2	30.60%	NR	NR
29. Kayaaslan et al, Turkey, 2021	To inquire about the presence of persistent symptoms beyond 12 weeks from the first diagnosis	Prospective Observational study	Questionnaire	August 1, 2020, and October 31, 2020	478	Fatigue, Myalgia, dyspnoea, Chest pain, Cough, palpations	median time 140 days	50 median (18 – 87)	48.10%	28	NR
30. Lund, et al, Denmark, 2021	To examine the occurrence of post-acute effects 2 weeks to 6 months after SARS-CoV-2 infection not requiring hospital admission	Retrospective cohort study	Clinical health records	Feb 27 and May 31, 2020	8983	Venous thromboembolism, dyspnoea needing bronchodilators	14 days – 180 days	43 median (30 – 56)	60.90%	NR	NR
31. Machado et al, Netherlands, 2021	To assess the construct validity of the PCFS Scale among adult subjects with confirmed and presumed COVID-19	cross-sectional study	Survey, Questionnaire, PCFS Scale, EQ-5D-5L, VAS, WPAI,	June 4th and June 11th, 2020.	1939	Fatigue, muscle weakness and sleeping problems were the most intense symptoms	mean 79 days	46 +/-11	85%	25.2 median (22.6 – 28.8)	6%
32. Mady et al, Egypt, 2021	To evaluate symptoms that persist after the acute stage of the disease in a cohort of patients with confirmed or	Retrospective study	EHR	N/A	143	Fatigue, Myalgia, dyspnoea, Cough	> 90 days	50 median (17 – 82)	45.10%	NR	28.7%

Author, Country, Year	Aim(s) of the study	Study Design	Data Collection protocol/ collection tool	Timeline of Data collection	Sample Size	Most common Symptoms	Days post infection	Average age	% of Women	AverageBMI	Smokers
	suspected COVID 19 and to define the predictors for long COVID syndrome.										
33. Mahmoud, et al, Saudi Arabia, 2021	To identify manifestations and predisposing factors for post-COVID-19 syndrome in Saudi Arabia.	Cross sectional observational study	Survey, questionnaire	May 2021 through June 2021	75	Dyspnoeaa, Fatigue, malaise, headache, mental and psychological problems	> 30 days	32.8 +/-5.2	57.30%	25.9 +/-3.21	75.30%
34. Matta, et al, France, 2021	To examine the associations of self-reported COVID-19 infection and SARS-CoV-2 serology test results in people with with persistent physical symptoms	Cross-sectional analysis	SAPRIS survey	December 2020 and January 2021,	25271	Sleep problems, joint pain, Back pain, muscle pain, fatigue, headache, breathing difficulty, chest pain, cough , dizziness	varied	49.9 +/-12.9	58.20%	NR	NR
35. Mittal,et al, India, 2021	To assess the prevalence and severity of Post COVID19 Symptoms and the treatment required and to make recommendation for Post COVID-19 care	cross sectional study	Medical notes, google forms self-documented from	NA	100	Weakness, Body ache Neuro-psychiatric symptoms restlessness and Giddiness, Respiratory symptoms, Headache, Palpitation,	42 – 84 days	57% 20-40 age group	40%	NR	NR

Author, Country, Year	Aim(s) of the study	Study Design	Data Collection protocol/ collection tool	Timeline of Data collection	Sample Size	Most common Symptoms	Days post infection	Average age	% of Women	AverageBMI	Smokers
						Chest pain, High B.P.					
36. Mohamed-Hussein, et al, Egypt, 2021	To assess long COVID-19 symptoms in hospitalised and non-hospitalised patients.	cross-sectional	Medical notes, online form, or interviews	1 July to 9 October 2020	167	Constitutional (fatigue), Respiratory, Neuropsychiatric	<84 days	38.7 +/-13.1	58.70%	27.2 +/- 7.8	16.20%
37. Munker, et al, Germany, 2021	To investigate and evaluate pulmonary function impairment after COVID-19 disease	Prospective Observational study	Pulmonary function impairment (PFI), DLCOcSB, TLC, FVC, or FEV1	March to August 2020	35	respiratory symptoms	120 days	43.3 +/- 17.4	62.90%	NR	14.30%
38. Naik, et al, India, 2021	To describe the clinical features and risk factors of post COVID-19 sequelae in the North Indian population	Prospective observational study	Interviews	October 2020 and February 2021	523	myalgia, fatigue, shortness of breath, dry cough	median 91 days	38.1	29.80%	NR	NR
39. Nehme, et al, Switzerland, 2021	To characterize symptoms 7 to 9 months after diagnosis of COVID-19	Prospective observational study	Self-reported surveys and semi structured telephone interviews	18 March and 15 May 2020	410	Fatigue, dyspnoea, Cough , Myalgia , chest pains	120 – 210 days	42.7	67.10%	NR	NR
40.Nielsen, K. J. et at, Denmark, 2021	To compare symptoms day by day for non-hospitalized individuals testing positive and negative SARS-CoV-2.	Prospective observational study	Questionnaire	March 11 and June 30, 2020	215	Dyspnoea, Cough, headache, sore throat, muscle pain,	90 days	30.5% between ages 40 -49	84.3%	NR	NR

Author, Country, Year	Aim(s) of the study	Study Design	Data Collection protocol/ collection tool	Timeline of Data collection	Sample Size	Most common Symptoms	Days post infection	Average age	% of Women	AverageBMI	Smokers
41. Ordinola Navarro, et al, Mexico, 2021	to evaluate the changes in quality-of-life (QOL) and spirometry alterations in the convalescent phase of 115 patients with at least 30 days post-COVID-19	Prospective observational study	EQ-5D-5L, Questionnaire VAS and spirometry	Apr 01 to Jul 30, 2020	115	Fatigue, dyspnoea, Cough, chest pains, Back pain	Median 30 days	40	49.50%	27.76	25.20%
42. Orrù, et al. Italy, 2021	To evaluate the physical and psychological health conditions of a representative sample of the Italian population suffering from symptoms related to long COVID.	cross-sectional	On online survey and ISI, EQ-5D questionnaires	5th February to 15 February 2021,	507	> 2 months reported the following symptoms: fatigue (79%), muscle aches/myalgia (53%), and headache (49%). > 3 months indicated that they suffered from these symptoms: fatigue (74%), muscle aches/myalgia (61%), and articular pains (59%)	30/60/ 90 days	30% of people between 40 - 49	82.05	NR	NR
43. Peghin, et al, Italy, 2021	To assess the prevalence of and factors associated with post-coronavirus disease 2019	Bidirectional prospective	interviews	September and November 2020	599	Fatigue, dyspnoea, Cough, joint pain, chest	Median 191 days	45.2	60.60%	14.9% obese	11.20%

Author, Country, Year	Aim(s) of the study	Study Design	Data Collection protocol/ collection tool	Timeline of Data collection	Sample Size	Most common Symptoms	Days post infection	Average age	% of Women	AverageBMI	Smokers
	(COVID-19) syndrome 6 months after the onset	cohort study				pains,neurological symptoms					
44. Petersen, et al, Faroe Island, 2021	To describe symptoms in the acute phase and especially long COVID in mainly non-hospitalized patients from the Faroe Islands.	Longitudinal study	Questionnaire, phone interviews. Self-reported physical performance loss, Likert QOL, and mental health and PHQ	3 March and 22 April 2020	180	Fatigue, dyspnoea , Cough , joint pain chest pains	Median 125 days	39.9	54%	NR	19.30%
45. Sahanic, et al , Italy, Austia, 2021	To explore recovery phenotypes in non-hospitalized COVID-19 individuals	Cross-sectional	online survey	30th September 2020 – 5th July 2021	4-12 weeks N= 990 <12 weeks N= 432	Fatigue, tired days, headaches, joint pain, dry cough, tachypnoea, chest pain , dyspnoea , bone pain, muscle pain	<90 days	44	66.70%	27.3	NR
46. Shendy, et al, Egypt, 2021	To determine the prevalence of fatigue in adult people post mild and moderate covid-19 case	Prospective observational study	Telephone questionnaire MFIS, Shortened FIM	15September 2020 - 15st December 2020	81	Fatigue	90 – 150 days	34	68%	29.6	NR
47. Shouman et	To identify the clinical features that overlap with symptoms of autonomic/small fibre	Retrospective cross-	medical record review,	March 2020 and	27	POTS Light-headedness, orthostatic	< 45 days	30	59%	NR	NR

Author, Country, Year	Aim(s) of the study	Study Design	Data Collection protocol/ collection tool	Timeline of Data collection	Sample Size	Most common Symptoms	Days post infection	Average age	% of Women	AverageBMI	Smokers
al,Germany, 2021	dysfunction and post covid syndrome	sectional study	Autonomic function testing	January 2021		headache, syncope, hyperhidrosis, and burning pain. Sudomotor function was abnormal, cardiovagal function, and cardiovascular adrenergic function.					
48. Sivan, et al, UK, 2021	To explore the presence of symptoms severity phenotypes in a community PCS cohort, including a large proportion of non-hospitalized participants, and understand the relationship between severity of symptoms, functional disability, and overall health in PCS	Cross sectional evaluation	C19-YRS	February 2 and May 3, 2021	370	Fatigue, anxiety, some pain or discomfort, breathlessness, some cognitive difficulties	median 211 day	47	64%	29	45%
49. Skala, et al, Czechia, 2021	To stratify post-COVID patients into four newly established clinical groups based on the presence or absence of at least one subjective respiratory symptom and at least one objective sign of pulmonary involvement	prospective study	Hospital records and clinical expert interviewer	June and October 2020 a	102	Fatigue, dyspnoea, cough, pain/chest discomfort, joint and muscle pain	90 days	46.7	54%	NR	NR

Author, Country, Year	Aim(s) of the study	Study Design	Data Collection protocol/ collection tool	Timeline of Data collection	Sample Size	Most common Symptoms	Days post infection	Average age	% of Women	AverageBMI	Smokers
50..Soraas,et al, Norway, 2021	To investigate the self reported health compared to one year ago three to eight months after COVID-19.	Prospective cohort study	Online survey, RAND-36	n February 1 and April 15, 2020	794	Fatigue, dyspnoea, cough, joint and muscle pain	90 days	26% ages 50 - 59	55%	NA	38%
51. Sudre, et al, UK, US and Sweden, 2021	To identify individuals at risk of long COVID	Prospective observational cohort study	Reporting on COVID Symptom Study app.		4182	fatigue, headache, dyspnoea, asomnia	Median 41 day	52	83.10%	26.5	NR
52. Sultana, et al, Bangladesh	To estimate the prevalence, length of illness, and risk factors of post-COVID symptoms	Descriptive cross-sectional analysis	A semi-structured questionnaire, telephone interviews, self-reported data	April 1, 2020, to July 30, 2020	186	fatigue, breathing difficulties, cough, palpations, chest pain, sleep disorders, muscle pain, headaches	30-60/ >61	34.8	33.90%	NR	13.40%
53. Tabacof, et al, USA 2022	To describe the persistent symptoms reported by a cohort of patients with PACS	Retrospective observational study	Surveys containing patient-reported outcomes	March 2020 and March 2021	156	Fatigue, dyspnoea, palpitations, muscle pain chest pains	90 days	44	69%	24	NR
54. Taquet, et al, UK, 2021	To describe the incidence, co-occurrence, and evolution of long-COVID features	Retrospective observational cohort study	EHR	December 2019 - December 16, 2020	106,578	Dyspnoea, fatigue/malaise, chest/throat pains, headache, other pain, myalgia	90 -180 days	39.4	58.40%	17.9% overweight	NR

Author, Country, Year	Aim(s) of the study	Study Design	Data Collection protocol/ collection tool	Timeline of Data collection	Sample Size	Most common Symptoms	Days post infection	Average age	% of Women	AverageBMI	Smokers
55. Townsend, et al, Ireland, 2020	To establish whether patients recovering from SARS-CoV-2 infection remained fatigued after their physical recovery, and to investigate whether there was a relationship between severe fatigue and a variety of clinicopathological parameters. We also sought to examine persistence of markers of disease beyond clinical resolution of infection.	Descriptive cross-sectional analysis	CFQ-11, EHR	NR	128	Physiological fatigue, physical fatigue	NR	49.5	43.90%	28.7	7.80%
56.Tran, et al, France, 2021	To develop and validate patient-reported instruments, based on patients' lived experiences, for monitoring the symptoms and impact of long coronavirus disease (COVID)	retrospective cohort study	Online survey with open ended questions with multiple-round qualitative content analysis to examine the content	14 October to 29 November 2020	1102	Fatigue, headaches, sleep disorders, and dyspnoea	Median time 217 days	45	84%	NR	NR
57. Vanichkachorn, et al, USA, 2021	To describe characteristics of a series of patients reporting prolonged symptoms after an infection with coronavirus disease 2019 (COVID-19)	A prospective observational study	medical record review and focused interview patient-reported history.		100	Fatigue, Dyspnoea, Cough , Headache Dizziness, Paraesthesia, Limits to ADLs	79.3 days	45.4	68%	30.2	NR

Author, Country, Year	Aim(s) of the study	Study Design	Data Collection protocol/ collection tool	Timeline of Data collection	Sample Size	Most common Symptoms	Days post infection	Average age	% of Women	AverageBMI	Smokers
58. Wang,et al, USA, 2021	To develop a comprehensive post-acute sequelae of COVID-19 (PASC) symptom lexicon (PASCLex) from clinical notes to support PASC symptom identification and research.	Longitudinal Study	EHR	March 4, 2020 and February 09, 2021	26,117	Pain, Insomnia, Anxiety, Pain in extremities, Depression, Paraesthesia, Fatigue, Peripheral oedema, Joint pain, Palpitations, Shortness of breath, Headache, Myalgia, Cough, Back pain, Weight gain, Pain in throat, Abnormal gait, Respiratory distress, Weight loss*, Dizziness or vertigo, Chest pain, Sleep apnoea, Weakness, Wheezing.	51 - 110 (a total of 60 days)	51.6	61.90%	NR	NR
59. Wanga, et al, USA, 2021	T0 recognize the most common post-COVID condition to optimize care and	Cross sectional study	Survey	January 2020–	698	Fatigue/Tired/ Weakness, Shortness of	30 days	26.3% = ages 20-29	48.50%	NR	NR

Author, Country, Year	Aim(s) of the study	Study Design	Data Collection protocol/ collection tool	Timeline of Data collection	Sample Size	Most common Symptoms	Days post infection	Average age	% of Women	AverageBMI	Smokers
	identify benefits of COVID-19 vaccination			April 2021		breath or breathlessness, Cough, Headache, Problems sleeping, Joint or muscle pain, Cognitive dysfunction, Chest pain or pressure, Post exertional malaise, Palpitations					
60. Yomogida, et al, USA, 2021	To identify of groups disproportionately affected by post-acute COVID-19 sequelae	A prospective observational study	EHDB, interviews, questionnaire	October 1, 2020–March 3, 2021	128	Fatigue, Dyspnoea, Cough, Headache, myalgia, or arthralgia	60 days	39% aged 25–39 years	56.60%	NR	NR

Supplementary Table 2: Percentages of Symptoms

	Article No.	Fatigue	Pain	SOB/ breathing disturbances	Headaches	Cough	Dizziness	Weakness	Palpations	Anxiety	Sleep disturbances	Orthostatic intolerance
Aly et al., Egypt 2021	31	8.3	19.6	20	5	6.67	ND	13.3	ND	ND	ND	ND
Augustin et al., Germany, 2021	32	9.3	2.6	8.6	ND	2.6	ND	ND	ND	ND	ND	ND
Bakilan et al., Turkey, 2021	33	12.1	4.6	ND	1.4	ND	ND	3.9	ND	ND	ND	ND
Barizien et al., France, 2021	34	30.7	0	ND	ND	ND	ND	ND	ND	ND	ND	ND
Bastola et al., Neapal, 2021	35	33.1	3.3	40.7	19.4	27.2	ND	ND	9.3	13.6	16.1	ND
Bierle et al., USA, 2021	36	73.8	90.2	42.9	ND	ND	ND	ND	ND	ND	ND	38.1
Bell et al., USA, 2021	37	37.5	0	37.5	ND	ND	ND	ND	ND	30.8	ND	ND
Bliddal et al., Denmark, 2021	38	15	16.5	11	4	4	ND	ND	ND	ND	ND	ND
Blomberg et al., Norway, 2021	39	30	11	15	ND	ND	10	ND	ND	ND	ND	ND
Boesl et al., Germany, 2021	40	62	83	ND	ND	ND	ND	ND	ND	ND	ND	ND

Buoite Stella, et al., Italy, 2021	41		24.9	27.7	ND	ND	5		ND	ND	ND	ND
Danesh et al., USA, 2021	42	52	24	73	ND	16	ND	ND	ND	ND	ND	ND
	Article No.	Fatigue	Pain	SOB/ breathing disturbances	Headaches	Cough	Dizziness	Weakness	Palpations	Anxiety	Sleep disturbances	Orthostatic intolerance
Davis et al., USA, UK, 2021	44	70.4	0	59.5	ND	ND	ND	ND	ND	ND	ND	ND
Dennis et al., UK, 2021	45	97.5	87.1	86.5	78.5	71.8	ND	ND	ND	ND	ND	ND
Gaber et al., UK, 2021	46	16.2	17.8	ND	ND	ND	ND	ND	ND	ND	9.9	ND
Ganesh et al., USA, 2021	47	ND	ND	ND	ND	ND	ND	ND	ND	ND	ND	ND
Gonzalez-Andrade, Ecuador, 2022	48	67.12	45.3	22.87	15.3	29.72	9.8	ND	12.88	26.6	36.48	ND
Graham et al., USA, 2021	49	85	43	47	37		47	ND	ND	ND	33	ND
Goertz et al., Netherlands, 2020	50	94.9	76	89.5	75.2	68.1	51.6	ND	54.9	ND	ND	ND
Heightman et al., UK, 2021	51	63.4	31.1	60.4	18.4	23.5	ND	ND	ND	ND	ND	ND
Herck et al., Netherlands, 2021	52	84.4	0	ND	ND	ND	ND	ND	ND	ND	ND	ND

Holmes et al., Australia, 2021	53	76.6	53.6	ND	21.4	64.3	ND	ND	ND	ND	ND	ND
Hossain et al., Bangladesh, 2021	54	82.9	0	ND	3	8.7	ND	ND	ND	ND	ND	ND
Jacobson et al., USA, 2021	55	29.5	0	21.1	14.7	8.7	ND	ND	ND	ND	ND	ND
Kamal et al., Egypt, 2021	56	72.8	34.1	28.2	28.9	ND	ND	ND	ND	38	ND	ND
	Article No.	Fatigue	Pain	SOB/ breathing disturbances	Headaches	Cough	Dizziness	Weakness	Palpations	Anxiety	Sleep disturbances	Orthostatic intolerance
Kayaaslan et al., Turkey, 2021	58	29.3	0	20.5	20.5	4.5	ND	ND	ND	ND	ND	ND
Lund et al., Denmark, 2021	59	ND	0	35.3	ND	ND	ND	ND	ND	ND	ND	ND
Mady et al., Egypt, 2021	60	16.8	10.5	6.3	ND	9.1	ND	ND	ND	21.7	ND	ND
Mahmoud et al., Saudi Arabia, 2021	61	72	48	85	ND	ND	ND	ND	ND	ND	ND	ND
Matta et al., France, 2021	62	2.5	16.5	0.8	0.6	0.6	0.6	ND	ND	ND	10.4	ND
Mittal et al., India, 2021	63		26	17	1		19	ND	14	ND	ND	ND
Mohamed-Hussein et al., Egypt, 2021	64	64.1	0	52.7	ND	ND	ND	ND	ND	ND	ND	ND

Munker et al., Germany, 2021	65		0	65.7	ND	ND	ND	ND	ND	ND	ND	ND
Naik et al., India, 2021	66	3.3	12.2	3.4	ND	1.7	ND	ND	ND	ND	ND	ND
Nehme et al., Switzerland, 2021	67	20.1	6.3	16.8	3.1	3.7	ND	ND	ND	ND	ND	ND
Ordinola Navarro et al., Mexico, 2021	68	25	0	15	19	10	ND	ND	ND	ND	ND	ND
Orrù et al., Italy, 2021	69	74	61	ND	ND	ND	ND	ND	ND	ND	ND	ND
	Article No.	Fatigue	Pain	SOB/ breathing disturbances	Headaches	Cough	Dizziness	Weakness	Palpations	Anxiety	Sleep disturbances	Orthostatic intolerance
Peghin et al., Italy, 2021	70	11	8	5.4	0.7	1.7	ND	ND	ND	ND	ND	ND
Petersen et al., Faroe Island, 2021	71	23.9	10	8	5	ND	ND	ND	ND	ND	ND	ND
Sahanic et al., Italy, Austia, 2021	72	34	15.5	13	13	3.7	ND	ND	33	ND	ND	ND
Shendy et al., Egypt, 2021	73	64.2	1.3	13	ND	ND	ND	ND	26.5	ND	ND	ND
Shouman et al., Germany, 2021	74		22	ND	ND	ND	93	ND	41	ND	ND	ND

Sivan et al., UK, 2021	75	95	0	85	ND	ND	ND	ND	ND	90	ND	ND
Skala et al., Czechia, 2021	76	22	3.9	23	13	13	ND	ND	ND	ND	ND	ND
Soraas et al., Norway, 2021	77	23	8	10	ND	8	ND	ND	ND	ND	ND	ND
Sudre et al., UK, US and Sweden, 2021	78	97.7	91.2	91.2	ND	ND	ND	ND	ND	ND	ND	ND
Sultana et al., Bangladesh	79	8.1	1.6	6.5	0.5	ND	ND	ND	ND	ND	3.8	ND
Tabacof et al., USA 2022	80	82	27	55	38	ND	ND	ND	45	ND	ND	ND
Taquet et al., UK, 2021	81	5.87	8.67	7.94	5.71	ND	ND	ND	ND	ND	ND	ND
Townsend et al., Ireland, 2020	82	11.38	0	ND	ND	ND	ND	ND	ND	7.8	ND	ND
	Article No.	Fatigue	Pain	SOB/ breathing disturbances	Headaches	Cough	Dizziness	Weakness	Palpations	Anxiety	Sleep disturbances	Orthostatic intolerance
Tran et al., France, 2021	83	87.9	69.3	55.7	ND	ND	ND	ND	ND	ND	59	ND
Vanichkachorn et al., USA, 2021	84	80	20	49	ND	15	19	ND	ND	ND	ND	ND
Wang,et al., USA, 2021	13	23.4	21	20.8	12.5	17.5	14	12.3	10.3	25.8	11.2	ND
Wanga et al., USA, 2021	86	24.7	7.3	15.5	12	13.8	ND	ND	ND	ND	ND	ND

Yomogida et al., USA, 2021	87	24.7	16.9	17.8	15.3	13.9	ND	ND	ND	ND	ND	ND

Key: ND = Not discussed
